# Roles and Clinical Applications of Biomarkers in Cardiovascular Disease 2017

**DOI:** 10.1155/2017/3093589

**Published:** 2017-10-17

**Authors:** Raffaele Serra, Stefano de Franciscis, Laurent Metzinger

**Affiliations:** ^1^Interuniversity Center of Phlebolymphology (CIFL), International Research and Educational Program in Clinical and Experimental Biotechnology, University Magna Graecia of Catanzaro, Viale Europa, 88100 Catanzaro, Italy; ^2^Department of Medical and Surgical Sciences, University of Catanzaro, Viale Europa, 88100 Catanzaro, Italy; ^3^C.U.R.S, Laboratoire INSERM U1088, Chemin du Thil, Université de Picardie Jules Verne, 80025 Amiens Cedex 1, France

Cardiovascular and Peripheral Vascular Disease (CPVD) is one of the major causes of mortality and morbidity worldwide.

Biomarkers can indicate several health or disease characteristics and they were found to be useful in order to improve diagnosis, prevention strategies, and treatment options in the field of CPVD. In fact, biomarkers may be used to better identify high-risk individuals, to diagnose disease conditions promptly and accurately, and to effectively prognosticate and treat patients with disease, targeting also specific biological sites.

As such, the present issue deals with both reviews and research articles focused on novel applications of biomarkers used to enhance the ability of the clinician to optimally manage the patient with CPVD. Furthermore, this special issue will discuss breakthrough biological and technological developments in biomarkers identification which are expected to revolutionize clinical research environments and healthcare in the area of CPVD.

The review by I. Szegedi et al. focused on biomarkers of atrial fibrillation and they based their study on four groups of biomarkers: markers of inflammation, markers of fibrosis, markers with hormonal activity, and other markers.

The review by L. Metzinger et al. considered the current evidences of epigenetic biomarkers dealing with the role of the main mechanisms of epigenetics in the area of cardiovascular risk. The main epigenetic processes showed in the review article are DNA methylation, posttranslational histone modifications, and RNA-based mechanisms such as noncoding RNAs, which are mainly represented by microRNAs (miRNAs), and long noncoding RNAs (lncRNAs).

The study by E. Aburto-Mejía et al. supported the idea that characterization of emerging biomarkers of impaired fibrinolysis such as plasminogen activator inhibitor type-1 (PAI-1) should be measured for surveillance of transition from a healthy state through the development of the Metabolic Syndrome to atherothrombotic disease.

The article by F.-Y. Chu et al. showed that high systolic and diastolic blood pressure variability may be correlated with the occurrence of peripheral arterial disease in the first decade following a diagnosis of type 2 diabetes mellitus.

The study by J.-R. Peng et al. showed that plasma levels of superoxide dismutases 1 and 2 (SOD1 and SOD2) were elevated in patients with coronary artery disease (CAD) and they concluded that these enzyme levels may be useful in the future as biomarkers for diagnosis of CAD.

The paper by J. Tian et al. presented a study on clinic predictive factors for insufficient myocardial reperfusion in ST-segment elevation myocardial infarction patients treated with selective aspiration thrombectomy during primary percutaneous coronary intervention showing the importance of identifying specific risk factors in order to improve the effectiveness of selective thrombus aspiration.

We hope that this special issue will attract the interest of scientific community in order to stimulate and improve further investigations leading to the discovery of novel biomarkers in the field of CPVD.

*Raffaele Serra* *Stefano de Franciscis* *Laurent Metzinger*

